# BIO::Phylo-phyloinformatic analysis using perl

**DOI:** 10.1186/1471-2105-12-63

**Published:** 2011-02-27

**Authors:** Rutger A Vos, Jason Caravas, Klaas Hartmann, Mark A Jensen, Chase Miller

**Affiliations:** 1 School of Biological Sciences, University of Reading, UK; 2 Department of Biological Sciences, Wayne State University, Detroit, MI, USA; 3 Tasmanian Aquaculture and Fisheries Institute, University of Tasmania, Australia; 4 Fortinbras Research, Rockville, MD, USA; 5 Center for Infection and Immunity, Columbia University, New York, NY, USA

## Abstract

**Background:**

Phyloinformatic analyses involve large amounts of data and metadata of complex structure. Collecting, processing, analyzing, visualizing and summarizing these data and metadata should be done in steps that can be automated and reproduced. This requires flexible, modular toolkits that can represent, manipulate and persist phylogenetic data and metadata as objects with programmable interfaces.

**Results:**

This paper presents Bio::Phylo, a Perl5 toolkit for phyloinformatic analysis. It implements classes and methods that are compatible with the well-known BioPerl toolkit, but is independent from it (making it easy to install) and features a richer API and a data model that is better able to manage the complex relationships between different fundamental data and metadata objects in phylogenetics. It supports commonly used file formats for phylogenetic data including the novel NeXML standard, which allows rich annotations of phylogenetic data to be stored and shared. Bio::Phylo can interact with BioPerl, thereby giving access to the file formats that BioPerl supports. Many methods for data simulation, transformation and manipulation, the analysis of tree shape, and tree visualization are provided.

**Conclusions:**

Bio::Phylo is composed of 59 richly documented Perl5 modules. It has been deployed successfully on a variety of computer architectures (including various Linux distributions, Mac OS X versions, Windows, Cygwin and UNIX-like systems). It is available as open source (GPL) software from http://search.cpan.org/dist/Bio-Phylo

## Background

Recent years have seen the emergence of the field of *phyloinformatics *[1]. At a practical level this is research where much of the organizational challenge lies in managing *data*, including character state matrices or multiple sequence alignments, phylogenetic trees and the relationships between these, and *metadata*, including cross references to molecular sequence databases, taxonomies, character state descriptions, biodiversity data, and literature references. At the nexus of the relationships between character state data and phylogenies lies the operational taxonomic unit (OTU), i.e. the biological entity on which observations are made (e.g. by measuring morphological traits or by sequencing DNA) and which is placed as a terminal node in a phylogeny.

In the course of a phyloinformatic analysis, data and metadata are collected or generated, transformed, filtered, analyzed and summarized before they can be interpreted to answer meaningful biological questions. Based on first principles of good science such steps should be reproducible; and, in practice, analysis steps often need to be redone by the researcher multiple times [2] and are too error-prone, tedious and time-consuming to perform manually. Hence, environments that allow such analyses to be scripted programmatically can greatly improve the efficiency and reproducibility of phyloinformatics.

Some of these facilities are provided by DendroPy [3], ETE [4] and BioPython [5] for the Python programming language, by the Ape package [6] for the R environment, and by BioPerl [7] for the Perl programming language. However, some of these (DendroPy, Ape), while strong on tree shape simulation and analysis, do not integrate easily in workflows that include external software for sequence alignment and phylogenetic inference or database or web service access, while others (BioPython, BioPerl, ETE) are strong in that respect but are lacking in tree shape simulation and analysis. In addition, none of these toolkits have a facility for managing the syntax and semantics of metadata. This can cause confusion when integrating and sharing metadata from disparate sources. For example, if an OTU is annotated with a taxonomic identifier, where (e.g. which database) does the identifier come from? What is the relation between the OTU and the database record (e.g. is the relationship established by a simple string match or something else)?

The Bio::Phylo toolkit addresses these issues, allowing researchers to read and write previously unsupported data formats, generate and transform data in a variety of ways, compute heretofore unimplemented topological indices, apply heretofore unavailable sampling and resampling algorithms and visualize the results in publication-ready graphics, while allowing phylogenetic knowledge to be managed, represented and shared in ways that preserve its meaning and its relation to metadata, regardless of its origin or context.

Due to its implementation in the Perl programming language and its compatibility with the BioPerl [7] toolkit, the operations supplied by Bio::Phylo are easily integrated in larger analysis workflows that take advantage of the operations supplied by BioPerl and that interface with command-line executables and web services, e.g. for database access or for computationally intensive analysis steps. However, BioPerl is very large and for many users difficult to install, whereas Bio::Phylo has no required dependencies. This makes deployment much easier for users who only require Bio::Phylo's functionality-orientated towards phyloinformatics per se-but not BioPerl's.

## Implementation

### Data and object model

**Figure 1:**
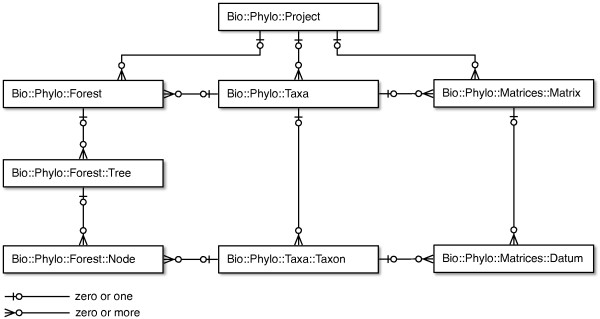
**Data model of core Bio::Phylo objects**. Cardinality relationships between the objects are shown as "crow's feet" notation; for example, a Bio::Phylo::Project has references to zero or more Bio::Phylo::Forest objects.

Bio::Phylo's design follows the data model shown in Figure 1. A phylogenetic project (Bio::Phylo::Project object) contains zero or more sets of trees (Bio::Phylo::Forest objects), zero or more sets of OTUs (Bio::Phylo::Taxa objects) and zero or more character state matrices (Bio::Phylo::Matrices::Matrix objects). Each forest object and each character state matrix may refer to a set of OTUs; however, this is not compulsory throughout the life cycle of these objects. For example, a tree parsed from a simple Newick [8] tree description contains terminal nodes-which may imply associated OTUs-but OTUs for these terminal nodes might only be instantiated when the tree is used in a context that explicitly requires them, such as when writing the tree to a file format that uses the OTU concept (e.g. as in NEXUS [9] "taxa blocks").

Each forest object contains zero or more tree (Bio::Phylo::Forest::Tree) objects, which contain zero or more nodes (Bio::Phylo::Forest::Node). Each of these nodes may have a reference to an OTU (Bio::Phylo::Taxa::Taxon) object, which, conversely, may have references to many nodes. For example, if a NEXUS file with multiple trees for the same set of species is read, the terminal nodes for the same species in the different trees will all hold a reference to the same OTU object, and that OTU object will hold references to all terminal nodes that reference it.

Each character state matrix contains zero or more datum (Bio::Phylo::Matrices::Datum) objects, which represent a character state observation. An observation could be a single character state-such as a morphological state-or a character state sequence, such as a DNA, RNA, amino acid, restriction site, categorical state or continuous state sequence. In addition to holding raw character state symbols, datum objects also manage the semantics of the data, e.g. which symbols are ambiguity symbols for sets of others (as per the IUPAC single character symbols [10]) including "missing" (which means an ambiguity symbol for the set of all possible states) and "gap" (which means an ambiguity symbol for the set of none of the possible states, i.e. "does not apply"). Each of these datum objects may reference an OTU object, which conversely may reference many datum objects.

OTU objects are used in Bio::Phylo to remap the relationships between tree nodes and datum objects from many-to-many to one-to-many from OTU to both nodes and data. This conceptualization is also implied in the NEXUS format and in software projects oriented towards data-management and data-exploration built on top of NEXUS such as Mesquite [11] and TreeBASE [12]. Similarly, containers of OTU objects (the Bio::Phylo::Taxa class) are implemented as objects from which one-to-many relationships exist to character state matrices and to sets of trees.

To implement the functionality implied by the data model (Figure 1) and described here, Bio::Phylo has been designed in object-oriented Perl 5, making use of abstractions and helper classes that have been omitted here for clarity, but which are documented exhaustively in the software release [see http://search.cpan.org/dist/Bio-Phylo].

### NeXML

All phylogenetic data objects in Bio::Phylo can be read from, and written to, NeXML http://www.nexml.org, a new XML format that is conceptually similar to NEXUS. The data objects all can hold references to zero or more Bio::Phylo::NeXML::Meta objects, which represent RDFa [13] annotations. This allows Bio::Phylo objects to be serialized as the subjects of "triples" [14] where the predicates and objects can be obtained from any controlled vocabulary or ontology (such as CDAO [15], SKOS [16] or DarwinCore http://rs.tdwg.org/dwc/index.htm), providing a flexible, semantic web-ready facility to attach metadata to phylogenetic data objects.

NeXML represents a considerable advance in the structured representation of phylogenetic data because these RDFa annotations allow phylogenetic data objects to be enriched with flexible predicates and objects, yet constrains these to explicit definitions in controlled vocabularies or ontologies. This is different from PhyloXML annotations and the NEXUS "notes block" because the predicates (or "keys", if viewed from the perspective of key/value annotations) in those formats are based on convention, not explicit definition, which is a situation that can cause ambiguities when integrating data from multiple sources.

A simple example may demonstrate this point: consider reading a file, matching the OTU names read from that file against names in the NCBI taxonomy, then sharing the results of this process. There is no non-ambiguous way to express in machine-readable form in NEXUS or PhyloXML why and how, for example, *Homo sapiens *became associated with the identifier 9606. Using NeXML with RDFa annotations, it can be expressed that the OTU with the name *Homo sapiens *(the subject of the triple) has a match, expressed using, for example, the *closeMatch *predicate from the SKOS vocabulary, with the taxonomy database record identified by http://purl.uniprot.org/taxonomy/9606. The definition of *closeMatch *in the SKOS vocabulary then clarifies unambiguously what the relationship is between the subject (the OTU) and the object (the database record). The key difference between this approach and others in use in phyloinformatics is the usage of formally defined predicates from any knowledge representation to describe the relation between objects and their annotations. (It is for this reason that the TreeBASE project [12] has adopted NeXML as its output format of choice to represent the wealth of metadata that TreeBASE contains.)

Due to NeXML's OTU-oriented data model, Bio::Phylo, which has a similar model, is a suitable target for NeXML I/O, whereas BioPerl, which lacks a notion of OTUs, is not. In addition, NeXML and Bio::Phylo support categorical and continuous character states, whereas BioPerl does not. By implementing Bio::Phylo with its level of support for NeXML, complex, richly annotated phyloinformatic data objects become amenable to processing in scriptable workflows while persisting more of their context and provenance than heretofore possible.

## Results and discussion

### Object manipulation and transformation

Bio::Phylo provides a toolkit for the manipulation of rich phylogenetic data objects. The objects can be annotated and labeled, and have any number of arbitrary other objects attached to them. The objects can be traversed in various ways, including depth-first, breadth-first or level-order traversal of tree shapes and through iterator or visitor access [17] to all objects that are lists of things (e.g. a Bio::Phylo::Taxa object is a list of OTU objects). Traversals can move from node objects to the OTU objects that define them (and back) and from OTU objects to character state observations that were made for the OTU objects (and back). The objects can be tested for various predicates, e.g. whether a tree is rooted, whether it is binary, whether it is ultrametric; whether a set of tips is monophyletic with respect to a given outgroup, whether a set of tips forms a complete clade; whether a node object is a tip, an internal node or the root node; whether it is the ancestor, parent, sibling, child or descendant of another node.

Using these traversal methods and tests, simple calculations are easily implemented. Bio::Phylo provides a number of these, e.g. the sum of all branch lengths on a tree; the average, minimum, maximum and cumulative root-to-tip path length; the amount of redundancy (i.e. the amount of shared, ancestral evolutionary history along all lineages on the total amount of evolutionary history, including along terminal branches). In addition, a number of more sophisticated tree shape methods useful for biodiversity informatics is provided:

▪ calc_ltt - Calculates lineage-through-time points [18].

▪ calc_symdiff - Calculates the symmetric difference metric [19] between two trees.

▪ calc_fiala_stemminess - Calculates stemminess measure [20].

▪ calc_rohlf_stemminess - Calculates stemminess measure [21].

▪ calc_imbalance - Calculates Colless's coefficient of tree imbalance [22].

▪ calc_gamma - Calculates γ-statistic [23].

▪ calc_i2 - Calculates I2 imbalance [24].

▪ calc_fp - Calculates the Fair Proportion value [25,26] for each tip.

▪ calc_es - Calculates the Equal Splits value [27,28] for each tip.

▪ calc_pe - Calculates the Pendant Edge [29] value for each tip.

▪ calc_shapley - Calculates the Shapley [30] value for each tip.

Likewise, calculations applicable to sets of trees (e.g. split frequencies) and to character state matrices (e.g. state frequencies, G/C content) are provided.

Bio::Phylo also provides methods for the transformation of phylogenetic data objects. For example, phylogenetic trees can be re-rooted, pruned or ultrametricized, nodes can be collapsed or inserted, branch lengths can be exponentiated or log-transformed. Sets of trees can be summarized in consensus trees or represented as pseudo-character-state MRP [31,32] matrices. Character state data can be manipulated directly, or transformed through bootstrapping and jackknifing [33].

### Input/output

A number of file formats is used for phylogenetic data. The Bio::Phylo::IO module supports the most commonly used ones: trees can be written and read in Newick format [8]; projects, taxa, trees and matrices can be written and read in NEXUS format and in NeXML http://www.nexml.org; character state matrices can be read from CSV, FASTA, PHYLIP and tab-delimited files; trees can be read from the Tree of Life Web Project [34] XML service; trees and character state matrices can be written in the legacy format for the CONTINUOUS [35,36], DISCRETE [37] and MULTISTATE [37] programs and in PHYLIP format

If BioPerl [7] is present, the wealth of data formats supported by Bio::SeqIO, Bio::AlignIO and Bio::TreeIO is also available because BioPerl objects can be converted to Bio::Phylo objects (using the new_from_bioperl constructors), and Bio::Phylo objects can be passed to the write methods of BioPerl. However, different from BioPerl is Bio::Phylo's concept of a "project" object, which is a collection of fundamental data objects (OTUs, trees and matrices) that reference each other. Whereas in BioPerl, NEXUS files are treated as flat containers of records of the same type (i.e. either trees or alignments, which are read sequentially by a tree file reader or an alignment reader, respectively), Bio::Phylo can optionally treat NEXUS and NeXML files as containing a project of related data of different types, in the same way as the informatics-oriented applications Mesquite [11] and TreeBASE [12] do. The compatibility with BioPerl is optional: Bio::Phylo doesn't require BioPerl to be installed or vice versa (they don't share code), but if Bio::Phylo detects BioPerl's presence, it enables a compatibility mode to make trees, nodes, character state matrices and sequences implement the interfaces that BioPerl defines.

Beyond BioPerl, interaction with other toolkits (e.g. ETE, DendroPy, BioPython, Ape) and combination in larger workflows is confined to the extent to which they can read the same data formats as Bio::Phylo. This functionality is usually confined to NEXUS and Newick file exchange, although DendroPy has support for NeXML as well, allowing more fine-grained data and metadata sharing, and similar functionality is in development for BioRuby [38].

### Visualization

**Figure 2:**
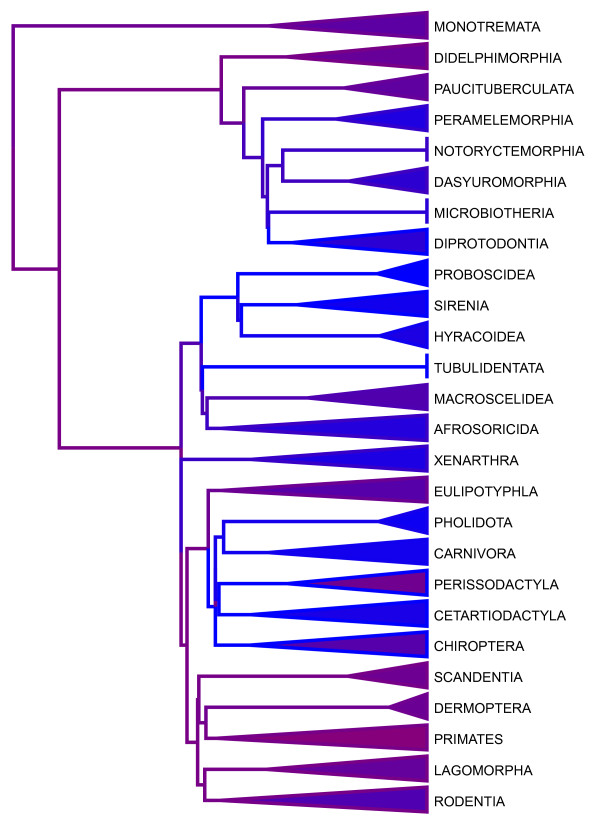
**Visualization example: mammal rates**. This tree was generated using Bio::Phylo by (i) reading a 3185 taxon phylogeny; (ii) collapsing the major clades; (iii) converting branch-specific speciation rates (read from a separate file, blue indicates low rates, purple and burgundy indicate higher rates) to RGB color codes; (iv) applying these colors to the branches and the average color within each collapsed clade to the triangles (data and use case kindly provided by Mark Pagel and Chris Venditti).

Bio::Phylo can draw trees where only the branching order and direction, but not the branch lengths are significant ("cladograms"), as trees with branch lengths proportional to time or some other metric such as inferred change ("phylograms") or as trees where branch lengths and distance are significant, but no direction or nesting is implied ("unrooted"). These trees can be drawn with rectangular, curved or diagonal branches. Branch thickness, branch color, node color, node radius and node pie diagrams (e.g. for "likelihood pies" [sensu [39]]) can all be set per node and branch individually. Clades can be represented in a view that shows them collapsed into triangles whose width and color can be set per clade individually. This programmatic access to the visualization of individual objects in large trees allows users to superimpose their data on trees in a variety of ways (for an example, see Figure 2).

The visualizations produced by the tree drawer module can be serialized to various bitmap formats (GIF, JPEG, PNG) and vector formats (PDF, SVG, SWF and the new HTML5 Canvas used by modern web browsers and by the iPhone and iPad), some of which can be used to create interactive graphics and animations (SVG, SWF and Canvas). Using the XML-based SVG output format, the resulting serialization can be further processed programmatically, as was done by the authors of a recent study [40] that used Bio::Phylo (Florent Angly, pers. comm.). Any serialization can of course be manipulated further by hand using vector drawing or graphics editing software to prepare it for publication; however, the most useful application of Bio::Phylo's tree drawing capabilities is probably in the creation of interactive graphics for the web, e.g. within the dynamic server environment of a web application that serves trees from a database.

### Sampling and simulation

Bio::Phylo can simulate tree topologies under the following models of cladogenesis: pure birth under the model of Hey [41]; pure birth under the Yule model [42]; equiprobable topologies [sensu [43]]; constant rate birth-death, evolving speciation rate and beta binomial models implemented using novel algorithms [44]. The tree sampling interface in Bio::Phylo can also be used to sample from arbitrarily complex user specified models using the algorithms in [44].

## Conclusions

Bio::Phylo is a software library written in Perl 5 that currently consists of about 33,000 lines of code spread out over 59 software modules. A manual that is part of the release provides the principal documentation. On a command line, this manual can be displayed by issuing the command perldoc Bio::Phylo::Manual. (The document is also available online http://search.cpan.org/dist/Bio-Phylo/lib/Bio/Phylo/Manual.pod). In addition, more documentation - about 14,000 lines as of revision 1204-can be found as embedded documentation in the individual classes of the release. For example, to learn more about reading and writing phylogenetic data, issue the command perldoc Bio::Phylo::IO. Bio::Phylo uses inheritance to a great extent, such that any one object may inherit additional methods from a number of super classes. In such cases, this will be noted in the "SEE ALSO" section of that class's documentation. The Bio::Phylo documentation system rewards the methodical reader who follows these document links.

Because Bio::Phylo implements the same interfaces in its core data objects, it is optionally compatible with BioPerl [7], filling a niche left open for phyloinformatic analysis in Perl. For example, the authors are aware of Bio::Phylo having been used for phylogenomic research [40], cladogenesis simulations [44], and the evaluation of biodiversity metrics [45]. Similar to Bio::NEXUS [46], Bio::Phylo implements more exhaustive implementation of the NEXUS data format than BioPerl does, however, Bio::NEXUS's functionality and use cases are confined to input and output in that format, omitting the object manipulation, annotation, visualization and sampling and simulation features discussed here.

**Figure 3:**
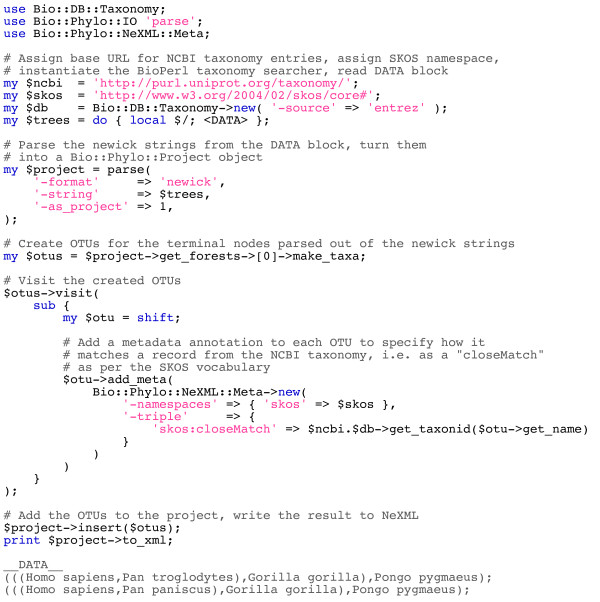
**Code sample: parsing OTUs from Newick tree descriptions and annotating them with NCBI taxonomy database record identifiers using the SKOS vocabulary to describe the relationship between the OTU and the database record (i.e. a close string match)**.

In comparison with open source projects for phyloinformatics implemented in other programming languages, Bio::Phylo most closely resembles the *ape *toolkit for R [6] and the *DendroPy *toolkit for Python [3], however, the language environments of these three projects are obviously different both in actual functionality and in their appeal to differing sense of aesthetics among user communities. Also, the compatibility with BioPerl and the integration with NeXML make Bio::Phylo especially suitable for applications where richly annotated phylogenetic data objects need to be persisted such that the semantics of the metadata are preserved (e.g. between steps in a larger workflow, or as data are serialized for sharing). A simple example of this is shown in Figure 3. In this code sample, two Newick trees are parsed; subsequently, OTUs for the terminal nodes in the trees are annotated with the *skos:closeMatch *predicate that describes the relationship between the OTUs and the respective NCBI taxonomy database records returned by the Entrez web service as best matches for the OTU names. In the final step of the example, the annotated project is persisted to NeXML with the metadata available to other consumers (such as a different script, or a client to a web service) and preserving in machine-readable format *why *the metadata exist.

### Future directions

Bio::Phylo is written in Perl, and while this language has many useful features, it is not very well suited for computationally intensive calculations compared to compiled languages such as C/C++. This means that operations such as maximum likelihood estimation or Markov chains are not usefully implemented in Perl, and so Bio::Phylo does not attempt this. However, Perl can easily make use of applications written in other languages through system calls, which is why it is used as a "glue language" for analysis workflows [2]. This is one of the main use cases for Bio::Phylo. In addition to writing one-off wrappers around command line tools, in this scenario one can also use BioPerl's *bioperl-run *extension, which Bio::Phylo can be seamlessly incorporated into because its objects can, optionally, double as the BioPerl objects that are passed into the wrappers that *bioperl-run *provides. However, this interface is somewhat coarse in the amount of allowed parameterization of wrapped applications, and so novel algorithms are generally difficult to implement this way. Several projects developed in compiled languages-for example HyPhy [47] and BEAGLE [48]-are now being designed so that they can be used as computational back-ends that can be connected to front-ends implemented in scripting languages such as Perl (e.g. using SWIG). Some preliminary tests have shown that it is possible to expose some of HyPhy's and BEAGLE's functionality to Bio::Phylo in this way, and this is something that will be explored further in the future, possibly as optional extensions to Bio::Phylo.

Other developments that might affect Bio::Phylo's future implementation and project organization are some of the new initiatives that are being undertaken in the Perl/bioinformatics community. The BioPerl project has grown to such a size that work is now being undertaken by its core developers to develop a more decentralized and modular architecture. If this results in a model whereby the different components in this new BioPerl ecosystem are decoupled enough that Bio::Phylo can participate in it while continuing to be developed along its original design and API it is the intention to make it part of this ecosystem. The recent decomposition of the BioPerl source code into multiple repositories maintained under the "git" revision control system simplifies this; recent discussions between BioPerl and Bio::Phylo developers point to a solution where Bio::Phylo becomes one such repository (e.g. "bioperl-phylo"). The hope is that this will ensure continued compliance of Bio::Phylo's implementations of BioPerl interfaces and that it will invite BioPerl users and developers to consider getting involved in Bio::Phylo where it fills niches left open by BioPerl. In fact, Bio::Phylo has been used successfully as an underlying engine for the parsing and production of NeXML format within the BioPerl core code base itself, through the module Bio::Nexml (written by CM). Bio::Nexml is an example of the transparent interoperability of the two toolkits, and a model of how continued cross-fertilization might proceed.

Yet other initiatives in the BioPerl community pertain to innovation in the Perl language itself. For Perl version 5, a new meta-programming extension called Moose greatly simplifies class definitions, and usage of this extension is being explored for BioPerl under the BIOME http://github.com/cjfields/biome project. It has been a guiding principle in Bio::Phylo's development that it does not require any prerequisites that are not part of the Perl core in order to be installed (although some functionality will be unavailable at runtime unless and until optional extensions are installed as specified in the 'Availability and Requirements' section). Since the Moose extension is currently not part of the Perl 5 core, redesigning Bio::Phylo along similar lines as the BIOME project would introduce a prerequisite to installing Bio::Phylo without adding any functionality. At present this makes this an unlikely scenario, but this might change depending on the success of BIOME and inclusion of Moose in the Perl 5 core.

Lastly, an important innovation is the development of the Perl 6 language http://dev.perl.org/perl6/. This new version of the language itself is a break with all preceding ones, with significant differences in syntax and in the object system. The new object system has several useful properties that will promote the development and maintenance of larger, more easily maintainable software projects. However, version 6 is so different from version 5 that adapting Bio::Phylo to it would amount to a port to a new language. Although this is currently being explored for BioPerl, the time investment is not justifiable for Bio::Phylo as long as Perl 6 virtual machines are not installed by default (or indeed available at all) on a great number of operating systems. This, however, is likely to change in the future.

## Availability and requirements

▪ Project name: Bio::Phylo

▪ Project home page: http://search.cpan.org/dist/Bio-Phylo/

▪ Source code repository: http://nexml.svn.sourceforge.net/svnroot/nexml/trunk/nexml/perl

▪ Operating system: platform independent

▪ Programming language: Perl

▪ License: GNU General Public License, version 3

▪ Other requirements: can all be installed from CPAN, either before or after installation of Bio::Phylo itself. These need to be present to activate certain functionality:

○ **Visualization **- any of the following to create the respective serializations: SVG.pm, SWF::Builder, PDF::API2, GD (for bitmap formats).

○ **Tree simulation **- Math::Random, Math::CDF

○ **NeXML I/O **- XML::Twig (for reading), XML::LibXML (optional, alternate way of creating DOM objects from Bio::Phylo objects).

### Installation

Bio::Phylo is installed like any other CPAN-compliant pure Perl distribution; it requires no compilation, but it does require the *make *(or on Windows, *nmake*) utility, which is freely available on all common operating systems. Bio::Phylo has been successfully installed on a variety of architectures. Installation testing reports collected by CPAN show successful installs on Linux (versions 2.6.26-2-686, 2.6.18-5-alpha-generic, 2.6.26-1-orion5x, 2.6.26-2-amd64 and 2.6.18-4-xen-amd64), Mac OS X (Darwin versions 8.11.0, 8.11.1 and 10.2.0, equivalent to OS X versions 10.4.x and 10.6.x), FreeBSD (versions 6.2-release, 6.4-release, 7.0-release and 8.0-release), NetBSD (version 4.0.1), IRIX (version 6.5), SunOS/Solaris (versions 2.9 and 2.11) and Windows (version 5.00, i.e. "Windows 2000" and Cygwin version 1.5.24(0.15642)). Perl versions upward from 5.8.x are known to work generally, with successful installs using 5.8.6, 5.8.7, 5.8.8, 5.8.9, 5.10.0 and 5.10.1 on threaded and unthreaded perls.

## List of abbreviations

API: Application Programming Interface; CPAN: Comprehensive Perl Archive Network; DOM: Document Object Model; GIF: Graphics Interchange Format; HTML: Hypertext Markup Language; JPEG: Joint Photographic Experts Group; PDF: Portable Document Format; PNG: Portable Network Graphics; SVG: Scalable Vector Graphics; SWF: Small Web Format (or ShockWave Flash); SWIG: Simplified Wrapper and Interface Generator; XML: eXtensible Markup Language

## Authors' contributions

RAV carried out the initial design of Bio::Phylo and drafted the manuscript. JC, KH, MJ and CM contributed selected parts of the code base and helped draft the manuscript. All authors read and approved the final manuscript.
